# Hyperglycemia and antibody titres against heat shock protein 27 in traumatic brain injury patients on parenteral nutrition

**Published:** 2014-02

**Authors:** Seyedeh Neda Mousavi, Fariborz Samini, Mohsen Nematy, Elena Philippou, Mohammad Safarian, Shima Tavallaiee, Abdolreza Norouzy

**Affiliations:** 1 Department of Nutrition, Endoscopic and Minimally Invasive Surgery, and Cancer Research Center, Mashhad University of Medical Sciences, Mashhad, Iran; 2 Department of Neurosurgery, Research Center of Orthopedic Surgery of Shahid Kamyab Hospital, Mashhad, Iran; 3 Department of Life and Health Sciences, School of Sciences and Engineering, University of Nicosia, Nicosia, Cyprus

**Keywords:** Glucose control, Heat shock protein 27, Parenteral nutrition, Traumatic brain injury

## Abstract

***Objective(s):*** Hyperglycemia worsens the neuronal death induced by cerebral ischemia. Previous studies demonstrated that diabetic hyperglycemia suppressed the expression of heat shock protein 70 and 60 (HSP70 and 60) in the liver. IgG antibody titres against heat shock protein 27 (anti HSP27) were measured to determine whether hyperglycemia exacerbates ischemic brain damage by suppressing the expression of heat shock protein 27 (HSP27) in the brain.

***Materials and Methods:*** A randomized controlled study of traumatic brain injury ICU patients treated either by intensive insulin treatment (IIT) or by conventional glucose control (CGC) was conducted. Patients received at least 50% of their estimated daily energy requirements parenterally. Serum anti HSP27 antibody concentration was assessed at baseline, day 7 and day 14.

***Results:*** Twenty-six out of 29 patients (n=13 in each group) completed the study. At baseline, there were no differences between the two groups. On day 14, there was a significant reduction in anti HSP27 titre concentration in the IIT compared to the GCG group (0.47±0.27 mg/dl vs 0.60±0.15 mg/dl, *P*=0.03).

***Conclusion:*** In this study, intensive control of traumatic brain injury patients on parenteral nutrition reduced anti HSP27 titre, possibly suggesting a reduction in stress.

## Introduction

Hyperglycemia induced by parenteral nutrition (PN) has been shown to exacerbate secondary brain injury and independently predicts poor neurologic outcomes (1, 2) in patients with severe traumatic brain injury. Cells respond to a variety of environm-ental stresses such as cerebral ischemia by expres-sing a family of proteins called the heat shock proteins (HSPs) (3, 4). HSP27 is a member of the small HSP (sHSP) family of proteins and has a molecular weight of approximately 27 KDa, however, it can form large aggregates of up to 800 KDa in the cytosol. In adults, HSP27 is expressed at high levels in several normal tissues including breast, uterus, cervix, placenta, skin, lung, heart and platelets. In addition to its role as a chaperone, HSP27 has also several other potentially important roles. Chaperones are proteins that assist the non-covalent folding or unfolding and the assembly or disassembly of other macromolecular structures, but do not occur in these structures when the structures are performing their normal biological functions and have completed the processes of folding and/or assembly. These include cell migration, apoptosis, protection against oxidative stress, endothelial barrier function and modulation of inflammation. Transient cerebral ischemia of either focal or global type enhances the HSPs gene expression and protein synthesis (3, 4). It has been speculated that the role of these proteins during ischemia is to suppress neuronal cell death by stabilizing denatured proteins and removing damaged proteins for degradation or by interfering with apoptotic pathways. The objective of the present study was to assess whether HSP 27 concentration is affected by manipulating blood glucose (BG) regulation in traumatic brain injury intensive care unit (ICU) patients.

## Materials and Methods

A randomized controlled trial aiming to compare intensive insulin treatment (IIT) and conventional glucose control (CGC) in adult patients admitted to ICU with traumatic brain injury was designed. Inclusion criteria were patients aged ≥18 years with a Glasgow Coma Scale 4-9 who received at least 50% of their estimated energy requirements (EER)ia PN. Patients with liver, kidney, heart, pancreatic failure or type 1 or type 2 diabetes were excluded from participation. The study was approved by the Bioet-hics Committee of Mashhad Medical University and was registered in the Iranian Randomized Control Trial studies (Registration number: IRCT201111 158108N1). Written informed consent or assent was provided by the participants or a close family member, before participation. 

The patients energy requirements were estimated as 25 kcal/kg ideal body weight (calcula-ted as explained below) as proposed by the ESPEN guidelines (5). The patients body height was estima-ted using the ulna length measurement (5) and the ideal body weight was estimated as (6):

IBW (male) = 50+(2.3 height [in inches]-60)

IBW (female) = 45.5+(2.3 height [in inches]-60)

The patients were hospitalized in ICU for at least 7 days before enrolment in the study. At baseline, demographic and clinical characteristics were obta-ined, including patient’s age, gender, weight and height as reported by a close family member, BG concentration, GCS and Acute Physiology and Chro-nic Health Evaluation II score (APACHEII) results and medication type and dose (including hypog-lycemic agents). 

Each patient randomly received either IIT defined as insulin infusion with cessation for at least 2 hr if BG concentration fell 75 mg/dl or CGC, provided by subcutaneous insulin infusion when BG ≥180 mg/dl. In the IIT group, BG was assessed immediately after infusion of 50 regular insulin units and at 2 hr intervals thereafter throughout the 24 hr period. Insulin infusion was discontinued if BG fell 75 mg/dl and re-assessed after 2 hr and was only re-started if BG was higher than 75 mg/dl. BG concentration of the CGC patients was assessed every 12 hr and insulin was injected subcutaneously aiming to maintain BG 180 mg/dl. In both groups, BG concentration was measured in capillary blood using the IME-DC glucometer (Germany). Reliability was assessed by assessment of a venous blood sample taken every 24 hr using the glucose-oxidase method. The coefficient of variation between the two methods was found to be 3.1%. 

Serum IgG antibody titres against heat shock protein 27 (anti HSP27) were assessed at baseline and on days 7 and 14 using an in-house ELISA assay. Microtitre plates (NuncMaxisorp, Nottingham, UK) were coated with 100 ng per well recombinant human HSP27 dissolved in 50 µl carbonate buffer pH 9.6 incubated for 18 hr at 4°C under humidified conditions. The wells were washed three times with PBS buffer. Non-specific binding was reduced by blocking each well with 2% goat serum in PBS and 250 µl was added to each well and incubated for 30 min at 37°C and 30 min at room temperature. Wells were then washed three times with PBS. Serum was diluted 1:100 with 2% goat serum in PBS and 100 µl was added to each well in duplicate and incubated for 30 min at room temperature. After washing (four times in wash buffer and two times in PBS), 100 µl peroxidase conjugated-goat anti human IgG (Sigma-Aldrich, Poole, UK) diluted 1:500 with 2% goat serum in PBS, was added to each well, and incubated for 30 min at room temperature. After washing, 100 µl of TMB substrate (100 µl of 6 mg/ml TMB in DMSO was added to 10 ml of 50 mM acetate buffer, pH 4.5, containing 3 µl H_2_O_2_) was added per well and plate incubated for 15 min in the dark at room temperature. The reaction was terminated by adding 50 µl HCl 2 M per well. Optical density at 450 nm was measured using a Lab system Reader MF Microtitre plate reader with a reference wavelength of 620 or 570 nm. The within-assay and between-assay precision was 3.5% and 5.2% respectively. 

Data were analyzed according to intention to treat. Continuous variables were analyzed using the unpaired student t-test if normally distributed or by the Mann-Whitney rank-sum test if not normally distributed. Categorical variables were analyzed using either the Fischer’s exact test or the chi-squared test. To assess the variations in serum HSP27 antibody titres over the 14-day period, repeated measures ANOVA test was used. Data are presented as mean ± standard deviation (SD). A two sided *P*-value of <0.05 was considered statistically significant.

## Results

A total of 29 patients, all male (IIT group: n=13, age: 31±11 yrs, CGC group: n=16, age: 36.6±13 yrs) were randomly assigned to one of the two treatment groups. Three out of 16 patients assigned to the CGC group expired during the first 5 days of admission. There were no differences in the baseline chara-cteristics of the two groups, including the severity of injury as well as the provision and composition of PN. (IIT group: 86.7±7% of EER via PN, CGC group: 85.5±7% of EER via PN, *P*=0.60). There was no difference between the groups in the dosage/kg body weight of hyperglycemia-associated medication provided (*P*=0.72). None of the patients suffered from pneumonia or acute renal failure. The mean blood glucose concentration at baseline was 205.5±66 mg/dl in the IIT group and 194.6±52 mg/dl in the CGC group (*P*=0.66) (Table 1). 

As shown in the Table 2, there were no differences in the concentration of HSP27 titre between the two groups on admission and day 7. On Day 14, the IIT group had a significantly lower concentration of anti HSP27 titre (*P*=0.03). In addition, comparison of the changes from baseline between the two groups showed that there were significant differences between the groups on Day 14 (*P*=0.02) (Table 2).

**Table 1:** Baseline characteristics of the intensive insulin treatment (IIT) group and the twice daily blood glucose control (TDGC) group

Variable	IIT group	TDGC group	*P*-value ^a^
	Mean ± SD	Mean ± SD	
Number of subjects	13	13	0.41
Age (yrs)	31.0 ± 11	36.6 ± 13	0.27
Ideal body weight (kg)	71.6 ± 6.5	72.2 ± 7	0.83
APACHE II score	13.4 ± 2	12.6 ± 3	0.08
Glascow Coma Scale (GCS)	7.3 ± 1	8.4 ± 2	0.14
Blood glucose concentration on admission (mmol/l)	11.4 ± 3.7	10.8 ± 2.9	0.66
% of estimated total energy requirements covered by:			
Parenteral Nutrition	86.7 ± 7	85.5 ± 7	0.60
Enteral Nutrition	13.3 ± 7	14.5 ± 7	0.60
Composition of Parenteral Nutrition:			
Dextrose (ml)	777.0 ± 268	912.5 ± 206	0.13
Aminoacid (ml)	615.0 ± 219	625.0 ± 223	0.90
Intra lipid (ml)	808.0 ± 194	812.5 ± 214	0.90

^a ^Comparison between IIT and TDGC group. Categorical variables were compared by Mann-Whitney test and continuous variables were compared by t-test

**Table 2:** Comparison of the anti HSP27 titre over the study period in each group

	ITT group			CGC group			Repeated measures ANOVA of differences between groups					
	Baseline	Day 7	Day 14	Baseline	Day 7	Day 14	Baseline		Day 7		Day 14	
							F	*P* -value	F	*P*-value	F	*P* -value
Anti HSP27 titre	0.62±0.27	0.62±0.28	0.47±0.27	0.68±0.22	0.57±0.15	0.60±0.15	0.47	0.49	2.02	0.17	5.2	0.03

ITT: Intensive insulim treatment; CGC: Conventional glucose control

## Discussion

This randomized control trial of insulin treat-ment in which patients with traumatic brain injury received at least 50% of their nutritional needs via parenteral rout, showed benefit in terms of anti HSP27 titres. Outcome including anti HSP27 titre was less in tight control group but this difference was not significant on day 7. Fourteen days after intervention, HSP27 reduced significantly in tight control group (*P*=0.03). Totally, only a few studies about glycemic control in trauma patients and anti HSP27 measurement have been published. Blood glucose level is one of the variables that accelerates neural death. Therefore, controlling this factor tightly, can lead to better outcomes in neural conditions. Another study (10) has detected changes of HSP gene expression after global cerebral ischemia using DNA microarray and measured HSP levels using western blot analysis and immunohisto-chemistry. These results showed that hyperglycemic ischemia for 15 min enhanced the mRNA expressions of hsp27, hsp70, hsp70, hsp90A and hsp90B in the neocortical tissues after 1 hr of reperfusion. Our results are in agreement with the previously publish-ed findings showing that the expression of hsp27 was significantly increased in brain after global and/ or focal ischemia followed by 3 hr reperfusion (7- 9).

To our knowledge, this investigation was the first pilot study in this area in Iran. The study had a homogenous population. The small sample was one of the limitations and so further data collection is warranted before reaching final conclusions. Increased levels of HSPs may suggest that hyperglycemia induces an enhanced stress response in neurons. IIT reduced neuron's stress by reducing the level of this protein. 

For future studies, we suggest that larger sample sizes are needed. Mortality and duration of ICU/ hospital stay as the major points in this population that need large sample size. Also, this method should be taken into consideration in other ICU hospitalized patients and all other patients that have hyperglyce mia in ICU. With more studies in this area, we can prepare a good scale for ICU hospitalized patients on PN.

## Conclusion

Blood glucose level is one of the variables that accelerates neural death. Parenteral nutrition, head injury, stress and ICU stay lead to hyperglycemia. HSP27 is a stress factor. Therefore, reducing this factor in traumatic brain injury patients is an indicator of stress reduction. In this study we showed that IIT (intensive insulin therapy) as the best method for glucose control can reduce stress by reducing Anti-HSP27 in serum in traumatic brain injury patients that receive PN. However, larger sample sizes are needed to reach a better conclusion. 
